# Supplementary material for: Distinct epigenetic landscapes underlie the pathobiology of pancreatic cancer subtypes
Source: Nat Commun. 2018 May 17;9:1978. doi: 10.1038/s41467-018-04383-6 (PMC5958058; doi:10.1038/s41467-018-04383-6)
Supplement: Supplementary file 1 — Supplementary Information [file 41467_2018_4383_MOESM1_ESM.pdf]

SUPPLEMENTARY INFORMATION

Distinct Epigenetic Landscapes Underlie the Pathobiology of Pancreatic Cancer Subtypes

Lomberk, *et al*

Supplementary Information Contains:

Supplementary Figures 1-9

Supplementary Tables 1-4

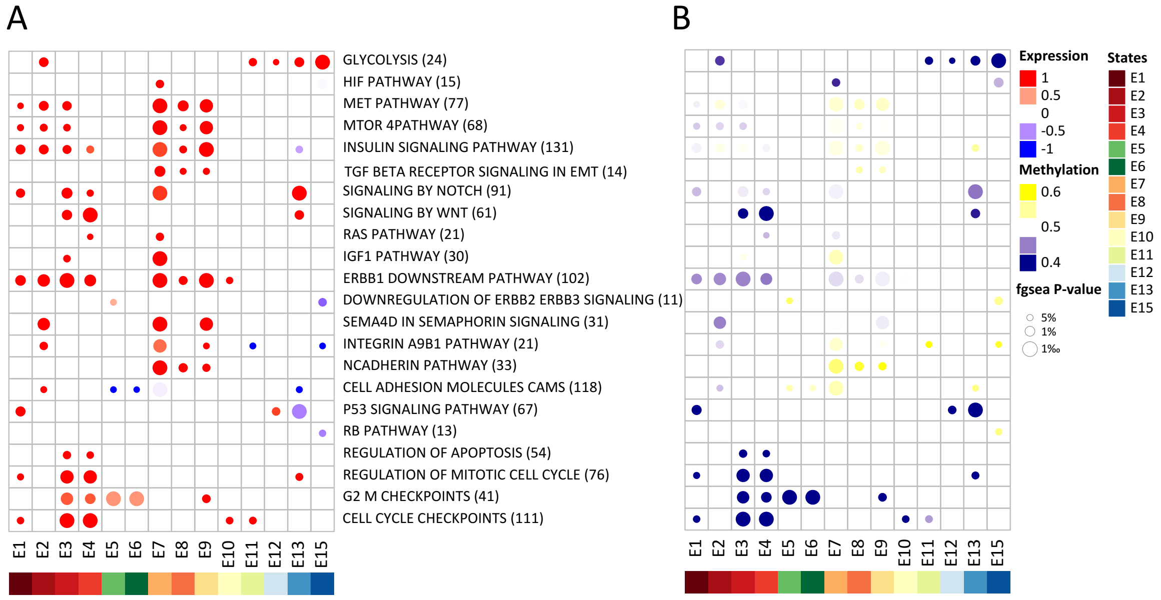

Supplementary Figure 1. Chromatin state enriched pathways

GSEA enriched pathways for each chromatin state. Circle size is proportional to $-\log_{10}$ p-value (showing only p-value<5%) and colors correspond in: (A) to the mean normalized expression of the leading-edge genes and (B) to the mean methylation level of the leading-edge genes.

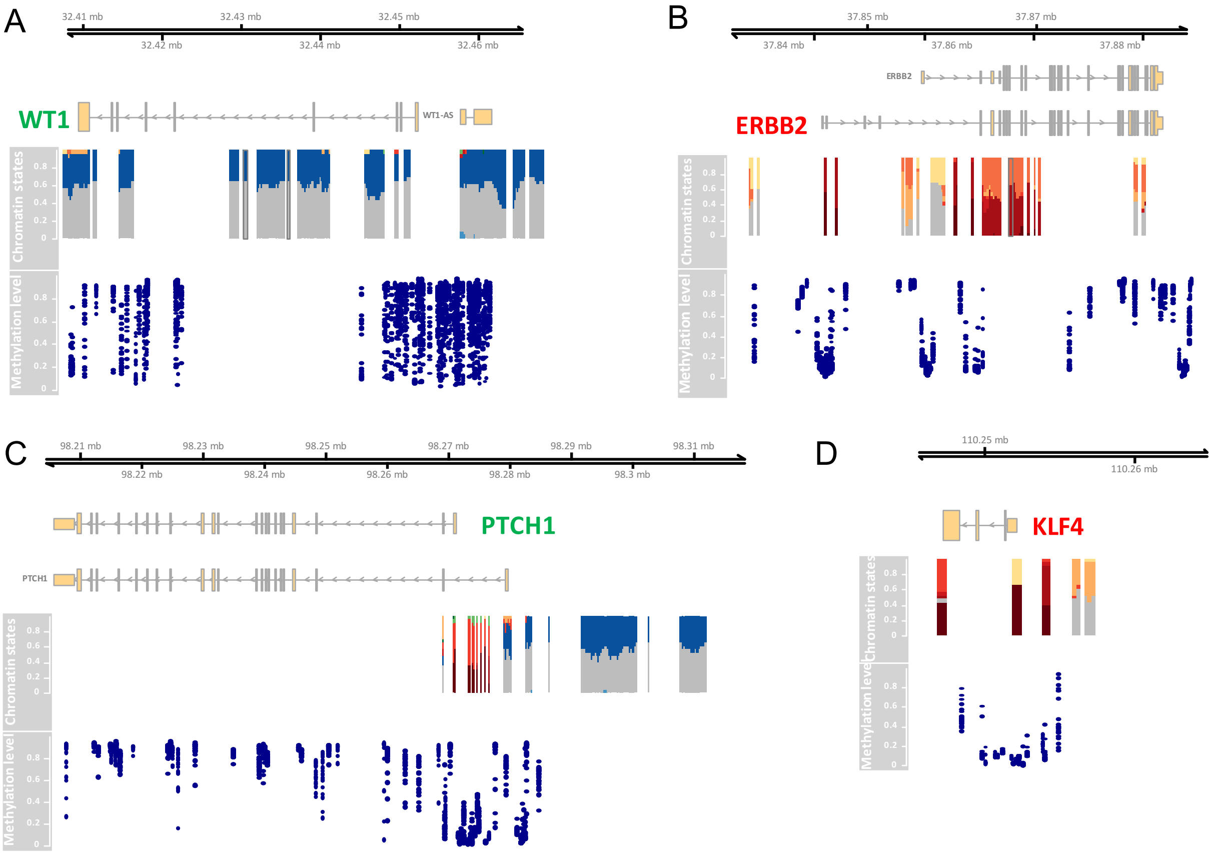

Supplementary Figure 2. Chromatin states at particular gene loci

Visualization of chromatin state proportions for all samples and methylation levels across the (A) SOX9, (B) ERBB2, (C) PTCH1 and (D) KLF4 loci.

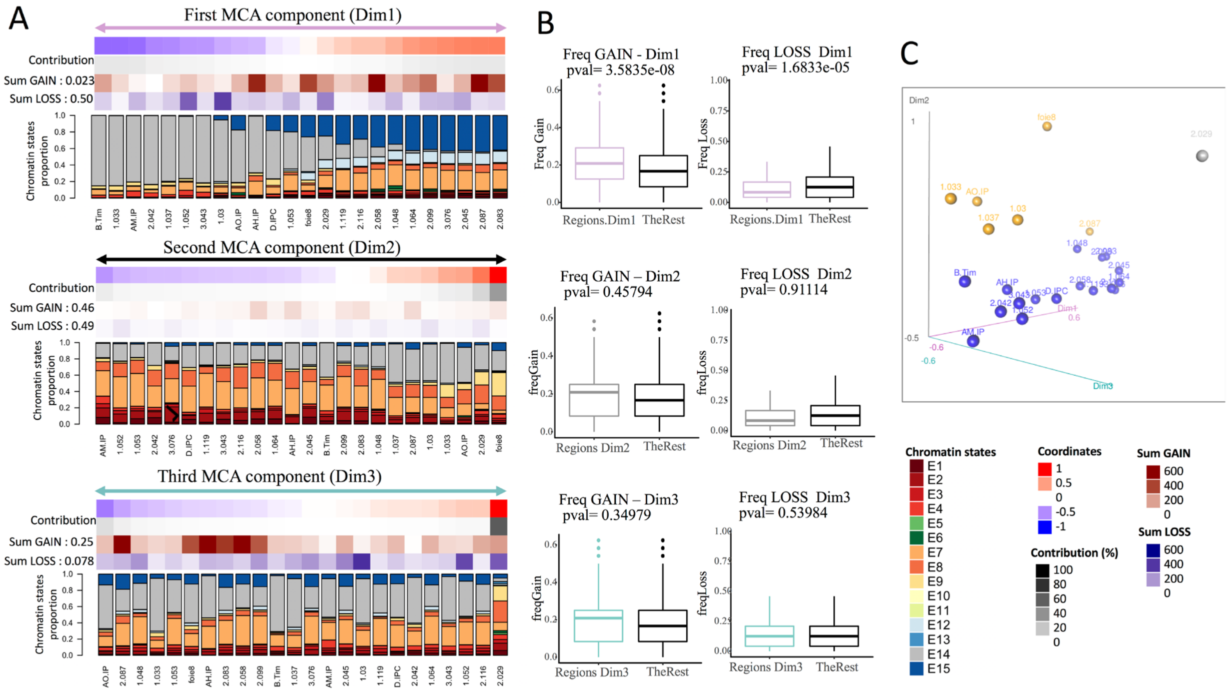

Supplementary Figure 3. MCA analysis

A. For each dimension, the sample coordinates (projection), the contribution (%), the sum of the component associated regions with are in gained or lost regions (SNP data), and a bar plot of the state's proportion calculated on the component associated regions are represented.

B. For each dimension, comparison of the gain or loss frequency for the component-associated regions compared to the one for the other regions are shown (Student's t test p-value). Bottom and top of boxplots are the first and third quartiles of the data, respectively, and whiskers represent the lowest (respectively highest) data point still within 1.5 interquartile range of the lower (respectively upper) quartile. Center line represents the median value.

C. 3D MCA plot. Dots are colored according to the chromatin-state based sample subtypes. The outlier is colored in grey.

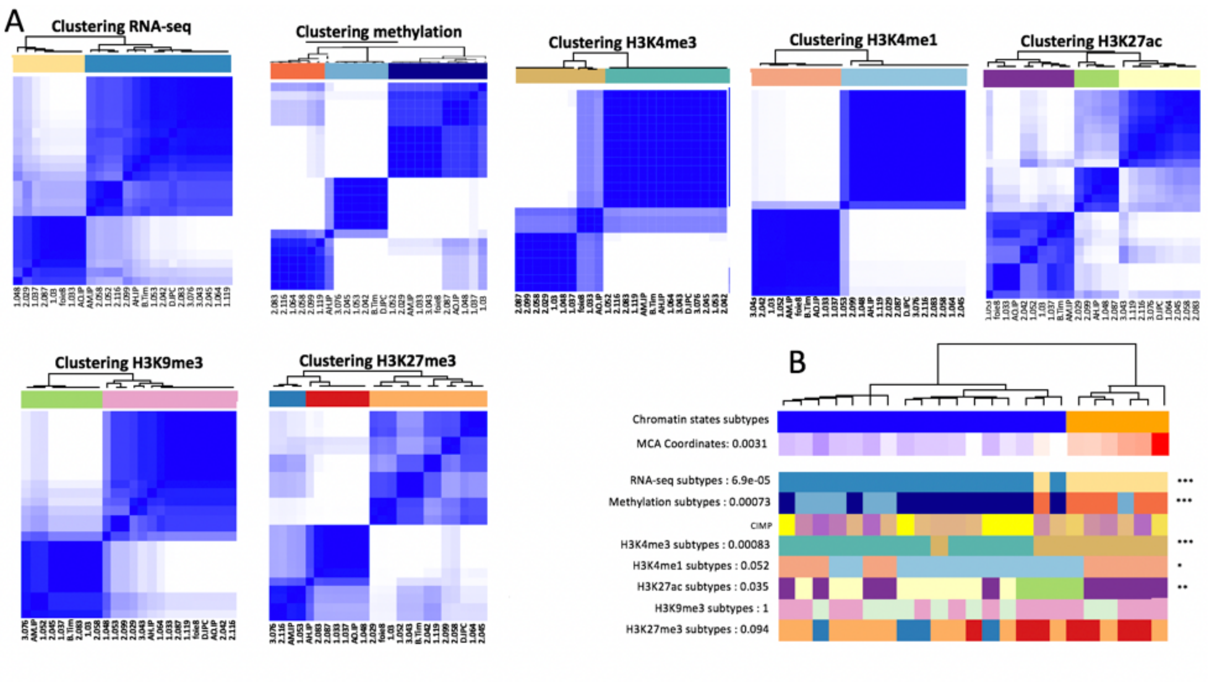

Supplementary Figure 4. Consensus clustering

A. Co-classification matrix resulting from consensus clustering analysis based on Histone marks, mRNA and methylation data. The strength of the blue color is proportional to the frequency at which samples have been clustered together. **B.** Comparison of the clustering with the chromatin state-based clustering (Fisher tests).

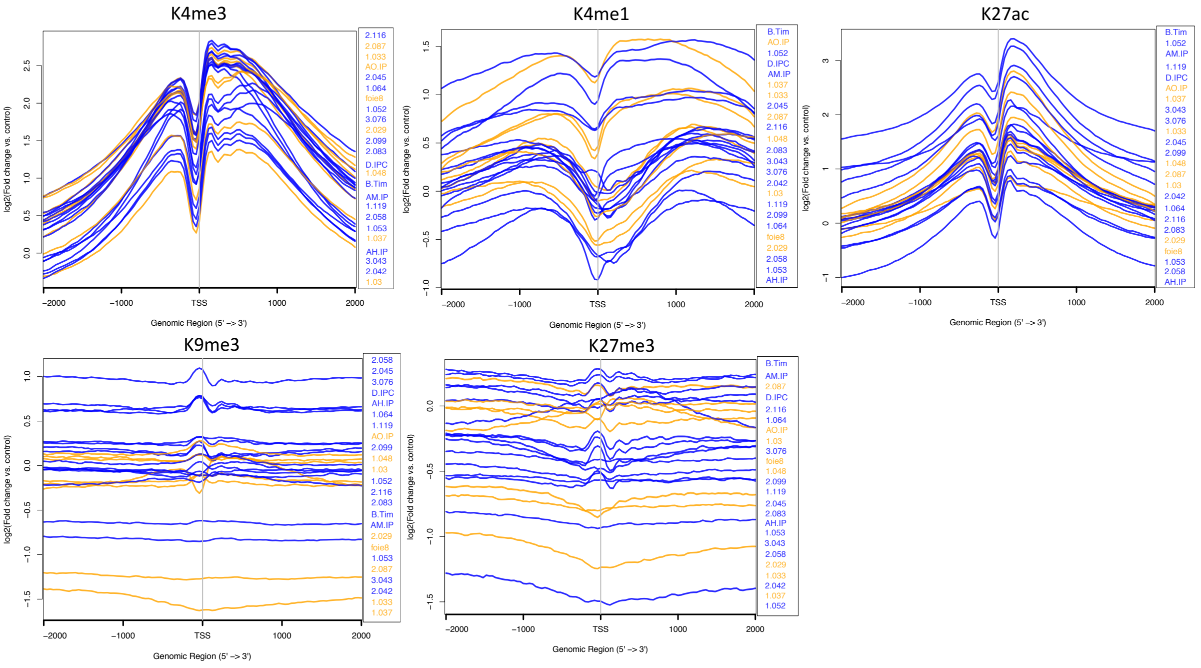

Supplementary Figure 5. Normalized read density across the TSS regions (+/-2000bp) for each histone modification in all PDAC samples. Basal tumors are plotted in orange and classical tumors in blue, demonstrating no distinct patterns in average profiles of these histone modifications for the two subtypes.

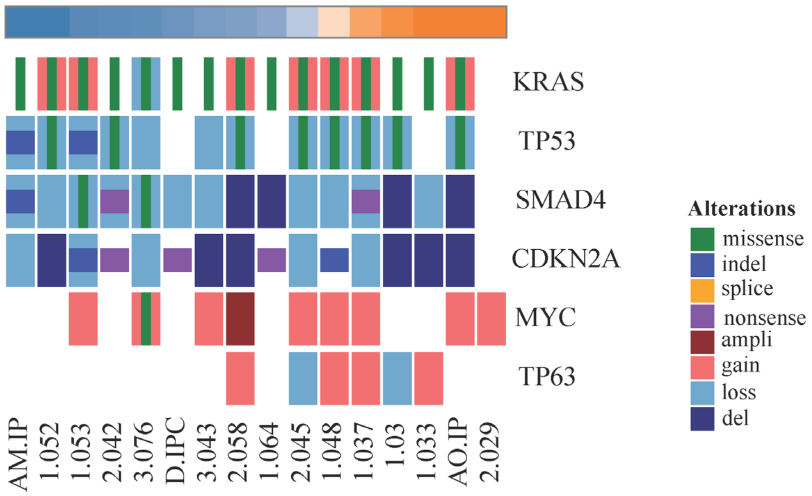

Supplementary Figure 6. Most frequent genomic alterations, including point mutations and copy number aberrations. The bar at the top indicates classical (blue) and basal (orange) tumors, which demonstrates that genomic alterations do not distinguish tumor subtypes.

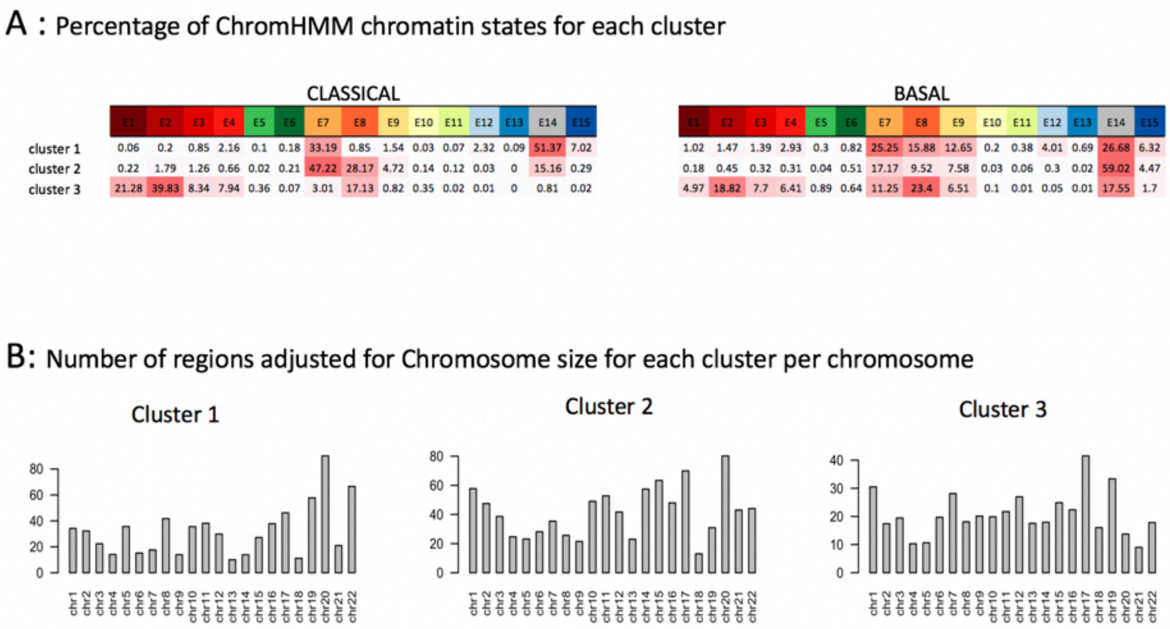

Supplementary Figure 7. Chromatin states clustering analysis and adjustment for chromosome size

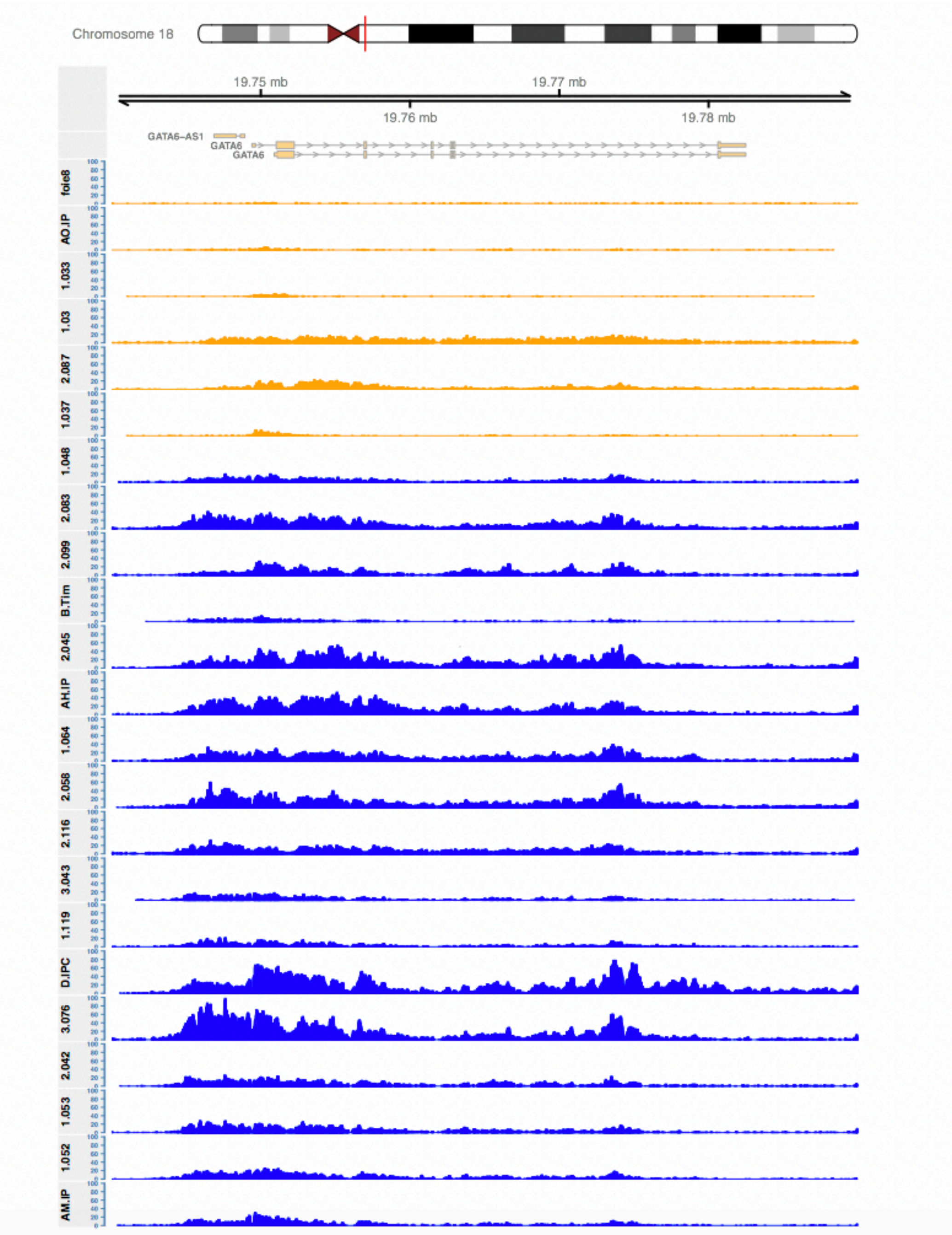

Supplementary Figure 8. GATA6 super-enhancer peak visualization for each sample.

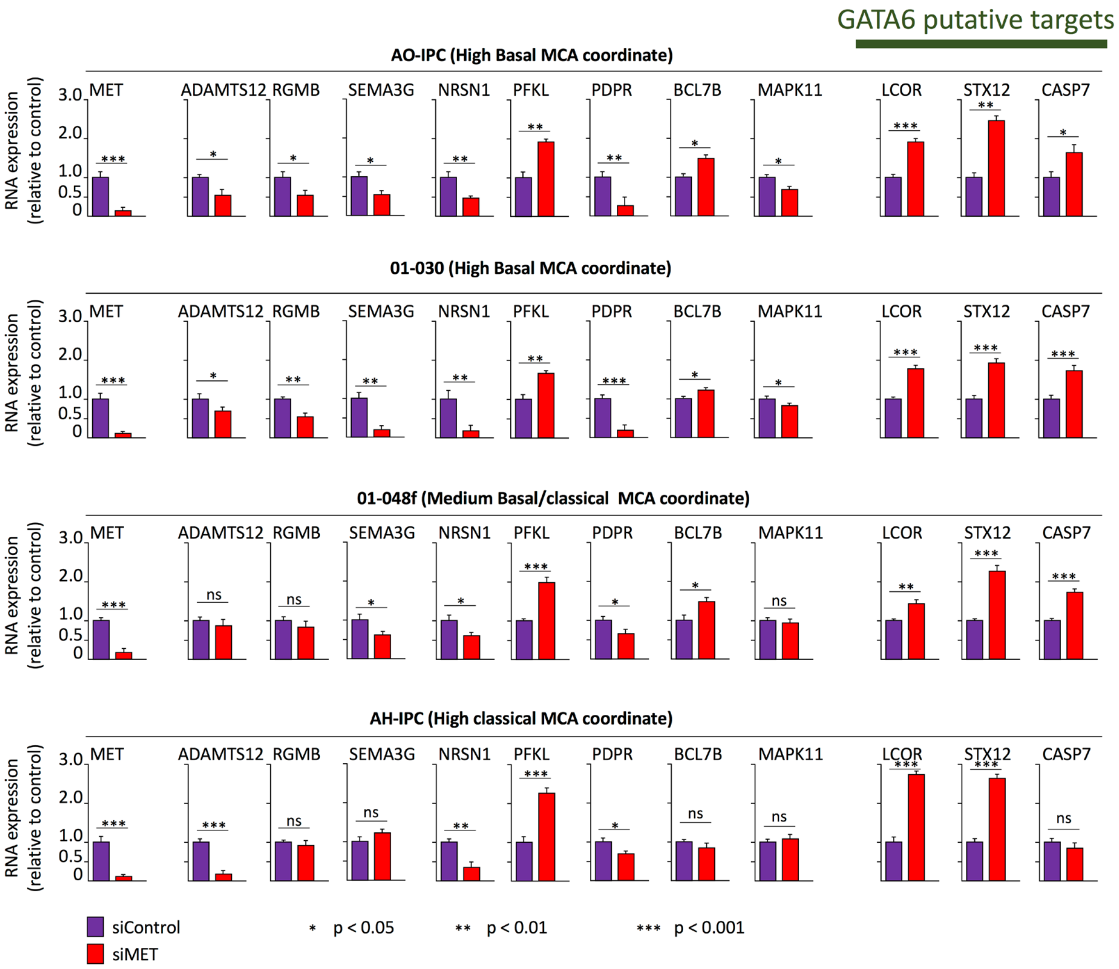

Supplementary Figure 9. qPCR validations on relevant biological markers. Error bars represent ± standard deviation. P-values as indicated on the figure were calculated by the Mann-Whitney two-tailed Test.

Supplementary Table 1: Clinical characteristics of the 24 PDTXs

ID.Consortium	Biomaterial.Collection.method	Age	Sex	Diagnosis	Diagnosis.Metastasis.Liver	Histology.Grade	TNM.Stage	Tumor.Location	Surgery.First	OS.event	OS.delay
1.03	Biopsy	66	F	Pancreatic Adenocarcinoma	1	G1	4	Pancreas	N	1	2
1.033	Biopsy	56	M	Pancreatic Adenocarcinoma	1	G3	4	Pancreas	N	1	3
1.037	Biopsy	58	M	Pancreatic Adenocarcinoma	1	G1	4	Pancreas	N	1	8
1.037	Biopsy	58	M	Pancreatic Adenocarcinoma	1	G1	4	Pancreas	N	1	8
1.048	Biopsy	64	F	Pancreatic Adenocarcinoma	1	NA	4	Pancreas	N	1	18
1.052	Biopsy	46	M	Pancreatic Adenocarcinoma	NA	G2	4	Pancreas	N	1	14
1.053	Surgery	63	M	Pancreatic Adenocarcinoma	0	G1	2b	Pancreas	Y	1	9
1.064	Surgery	67	F	Pancreatic Adenocarcinoma	0	G2	2b	Pancreas	Y	0	28
1.119	Surgery	81	M	Pancreatic Adenocarcinoma	NA	G2	2b	Pancreas	Y	1	10
2.029	Surgery	68	F	Pancreatic Adenocarcinoma	0	G2	2b	Pancreas	Y	0	39
2.042	Surgery	52	M	Pancreatic Adenocarcinoma	0	G1	2b	Pancreas	Y	1	18
2.045	Surgery	79	M	Pancreatic Adenocarcinoma	0	G1	2a	Pancreas	Y	1	12
2.058	Biopsy	78	F	Pancreatic Adenocarcinoma	0	NA	NA	Pancreas	N	1	10
2.083	Biopsy	74	F	Pancreatic Adenocarcinoma	1	NA	4	Pancreas	N	1	6
2.087	Biopsy	57	M	Pancreatic Adenocarcinoma	NA	NA	NA	Pancreas	N	1	12
2.099	Surgery	66	F	Pancreatic Adenocarcinoma	NA	G2	2b	Pancreas	Y	1	14
2.116	Surgery	69	F	Pancreatic Adenocarcinoma	0	G1	4	Pancreas	Y	1	5
3.043	Surgery	59	M	Pancreatic Adenocarcinoma	0	G1	2b	Pancreas	Y	1	10
3.076	Biopsy	61	M	Pancreatic Adenocarcinoma	1	NA	4	Pancreas	N	1	6
AH-IPC	Surgery	74	M	Pancreatic Adenocarcinoma	0	G2/G3	2b	Pancreas	Y	1	11
AM-IPC	Surgery	69	M	Pancreatic Adenocarcinoma	0	G3	2b	Pancreas	Y	0	33
AO-IPC	Surgery	63	F	Pancreatic Adenocarcinoma	0	G2	2b	Pancreas	Y	1	6
D-IPC	Surgery	50	F	Pancreatic Adenocarcinoma	0	G2	2b	Pancreas	Y	1	29
Foie8b	Biopsy	52	F	Pancreatic Adenocarcinoma	1	NA	4	Liver	N	1	3

Supplementary Table 2: Epigenetically deregulated pathways

	Top pathways	P-val	Genes
Cluster 1	epithelial cell differentiation (GO:0030855)	1.09E-04	YAP1;LAMA5;RXRA;MREG;HEY1;ITGA2;MYO5A;NF2;STK4;RHCG;E2F7
	regulation of cell division (GO:0051302)	3.02E-04	IL1A;PRKAR1A;IL1B;MYC;TGFA;RCC1;SFN;PGF;E2F7
	regulation of apoptotic signaling pathway (GO:2001233)	4.34E-04	YAP1;MLLT11;IL1A;NRP1;KDM1A;IL1B;PMAIP1;SFN;STK4;S100A9;CD44
	Hippo signaling pathway (hsa04390)	5.11E-04	YAP1;PPP2CA;WWTR1;MYC;WNT7B;FZD6;NF2
	TGF-beta signaling pathway (hsa04350)	7.27E-03	PPP2CA;SMURF2;MYC;FST
	Wnt signaling pathway (hsa04310)	9.72E-03	MYC;SERPINF1;WNT7B;RUVBL1;FZD6
	PI3K-Akt signaling pathway (hsa04151)	1.31E-02	PPP2CA;LAMA5;RXRA;MYC;ITGA2;MET;EGFR;PGF
	ErbB signaling pathway (hsa04012)	4.51E-02	MYC;TGFA;EGFR

	Top pathways	P-val	Genes
Cluster 2	positive regulation of insulin secretion (GO:0032024)	2.86E-06	PFKFB2;TCF7L2;CASR;RBP4;PDX1;ARRB1;MCU
	lipid biosynthetic process (GO:0008610)	1.15E-05	ACHE;ACSS2;PGAP3;SRD5A3;MGST2;PLA2G4A;ACSL5;TFCP2L1;PRLR;PRKAB1;CYP27A1;HSD11B2;HSD17B2;PLBD1;PIP5K1B;ANG;DEGS2;MPPE1;MGLL
	glandular epithelial cell differentiation (GO:0002067)	9.27E-05	BMP2;GATA6;PDX1;SPDEF
	Maturity onset diabetes of the young (hsa04950)	3.88E-03	NR5A2;HNF4A;PDX1
	Insulin secretion (hsa04911)	3.99E-03	PLCB4;CREB3L1;PDX1;ATP1B1;ADCY6
	Steroid hormone biosynthesis (hsa00140)	5.64E-03	HSD11B2;SRD5A3;HSD17B2;CYP3A5
	Gastric acid secretion (hsa04971)	1.31E-02	PLCB4;KCNK10;ATP1B1;ADCY6
	Hedgehog signaling pathway (hsa04340)	2.36E-02	BMP2;SHH;IHH

	Top pathways	P-val	Genes
Cluster 3	Maturity onset diabetes of the young (hsa04950)	4.26E-04	HNF1B;HNF1A;FOXA3
	hexose metabolic process (GO:0019318)	7.13E-04	HKDC1;LRP5;FUT2;FBP1;FUT4;ATF3
	endocrine pancreas development (GO:0031018)	1.42E-03	ONECUT2;HNF1B;FOXA3
	Steroid biosynthesis (hsa00100)	5.72E-03	CYP51A1;FDFT1
	Ras signaling pathway (hsa04014)	9.74E-03	EFNA1;PLA2G4F;KITLG;EFNA2;RASA3
	Fructose and mannose metabolism (hsa00051)	1.43E-02	HKDC1;FBP1
	Axon guidance (hsa04360)	3.61E-02	EFNA1;EFNA2;SEMA4G

Supplementary Table 3: Differentially expressed genes (DEG) between basal siMET and siControl (scrambled siRNA) samples

geneSIGN	p-value	t
RP11-1L12.3	1.95E-06	-41.860798
RP1-239B22.5	1.54E-03	7.69154
RGMB	2.00E-03	7.169738
TPM4	2.12E-03	-7.062344
HSDL2	2.28E-03	-6.930092
IFT43	3.68E-03	-6.089852
GANAB	3.81E-03	-6.031596
BICD2	4.08E-03	-5.919077
ZSCAN21	4.48E-03	5.7694
MET	5.78E-03	5.378017
LCOR	5.83E-03	-5.363568
COMMD1	6.44E-03	5.217584
GS1-44D20.1	7.30E-03	-5.035843
KLHDC3	8.49E-03	-4.824697
RMND5A	9.05E-03	4.737616
ASB3	1.01E-02	4.591216
HESX1	1.06E-02	-4.527169
RP5-1057J7.6	1.06E-02	4.521886
JPX	1.23E-02	-4.331092
ZNF551	1.24E-02	-4.321959
ZNF766	1.26E-02	-4.305729
AC120194.1	1.35E-02	4.215367
EEA1	1.38E-02	-4.192762
MTFR1L	1.48E-02	4.105075
PREPL	1.49E-02	4.096972
GPR75-ASB3	1.55E-02	4.049169
HCP5	1.62E-02	3.993808
STX12	1.63E-02	-3.988203
RP11-219B4.5	1.73E-02	3.915472
BAP1	1.80E-02	3.872624
IGIP	1.82E-02	3.855173
C9orf91	1.97E-02	3.766235
RP1-1J6.2	2.00E-02	-3.748437
FAM169A	2.03E-02	3.728222
SLC3A1	2.06E-02	3.712597
BTBD9	2.19E-02	3.642984
AC006449.1	2.20E-02	3.637264
EMC6	2.45E-02	-3.51562
C5orf55	2.53E-02	-3.481552
IL11RA	2.54E-02	3.476316
RPS4XP8	2.55E-02	3.475708
GPR83	2.64E-02	3.436849
ZNF605	2.71E-02	-3.408364
PRKAB1	2.82E-02	3.364183
SPSB3	2.83E-02	3.358656
FOXP4	2.85E-02	-3.353784
ANKRD52	2.97E-02	3.310073
TNFRSF21	3.25E-02	3.214313
AC004490.1	3.43E-02	-3.155078
PRDX4	3.44E-02	-3.154749
ARL5B	3.49E-02	3.139711
OSGIN1	3.56E-02	3.118922
TRAM1	3.58E-02	3.111726
AL391152.1	3.63E-02	-3.096749
RP1-317E23.7	3.65E-02	3.091424
NCR3LG1	3.74E-02	3.068206
RP1-317E23.6	3.78E-02	3.055373
MTSS1L	3.83E-02	-3.041741
PHACTR2	3.90E-02	-3.024249
PANK1	4.01E-02	-2.995802
AC133528.2	4.02E-02	-2.994419
RNF149	4.15E-02	2.962147
AC068580.7	4.21E-02	2.947394
DDTL	4.24E-02	2.939998
ZNF267	4.38E-02	-2.907419
SNX13	4.43E-02	2.89497
FLJ00418	4.49E-02	-2.882334
IGBP1-AS2	4.53E-02	2.87359
PBDC1	4.57E-02	-2.864794
MAP3K2	4.78E-02	-2.821405
RP11-485G4.2	4.88E-02	-2.800172
GRB10	4.89E-02	-2.797609
RPS23P8	4.94E-02	2.788195
RP11-697E22.1	1.39E-01	3.012046
RP11-841O20.2	2.19E-01	-3.802259
RP11-140K17.3	3.15E-01	-4.121427
RP11-101E13.5	5.42E-01	2.945923
bac-BPG157A10.21	3.34E-02	3.185579

Supplementary Table 4: Primer sequences for qPCR validation

Supplementary Table 4: Primer sequences for qPCR validation			
	Forward Primer (5'->3')	Reverse Primer (5'->3')	amplicon size
**MET**	AGCAATGGGGAGTGTAAAGAGG	CCCAGTCTTGTACTCAGCAAC	198
**RGMB**	TGTTGGGTATCAGTGACCTCA	CTTCGGGGTTGGTAGAGGATG	75
**STX12**	TGCAACAGTTACAACACTCCAC	GGCAAGGACCCTAATTCTTTCAG	76
**LCOR**	CTTCGGAGTGGTGATGGGGTA	GTGGAACTTTGAGTGATGTGGA	88
**PFKL**	GTACCTGGCGCTGGTATCTG	CCTCTCACACATGAAGTTCTCC	100
**PDPR**	TGCTGTCGATTGTTGGAAGAC	CACAGAAGTGCCCGTGATTC	155
**SEMA3G**	CGGGTGCTGGTGAACAAATG	CTCTAGCTGGTCAAAGTGGGT	99
**NRSN1**	TTTTGTGATCCTCGGATTGACTG	CAGAGCACTGTTAAACTGGACA	124
**CASP7**	AGGGACCGAGCTTGATGATG	CACTGGGATCTTGTATCGAGGA	85
**BCL7B**	TCCCTGAGGATATTTAAGTGGGT	CTCGGGCTGCTGAACTGTT	82
**ADAMTS12**	ACTTTGGGGCGCTTTGCTAT	GCAGGCCCTTGATAAAATGCT	90
**MAPK11**	AAGCACGAGAACGTCATCGG	TCACCAAGTACACTTCGCTGA	82
