# Supplementary material for: Distinct epigenetic landscapes underlie the pathobiology of pancreatic cancer subtypes
Source: Nat Commun. 2018 May 17;9:1978. doi: 10.1038/s41467-018-04383-6 (PMC5958058; doi:10.1038/s41467-018-04383-6)
Supplement: Supplementary file 3 — Description of Additional Supplementary Files [file 41467_2018_4383_MOESM3_ESM.pdf]

Description of Additional Supplementary Files

File Name: Supplementary Data 1
Description: Excel workbook listing the GSEA enriched pathways identified for each chromatin state (E1-E15) in separate spreadsheets. Gene score used in fgsea was the frequency among all samples of the presence of a particular chromatin state within -20kb from TSS +1kb from TSE of the gene. Leading edges correspond to genes that drive the enrichment as given by fgsea.

File Name: Supplementary Data 2
Description: Excel spreadsheet listing the GSEA enriched pathways for the siMET condition. Enrichment tests were performed based on P-values of the differential RNA-seq analysis of basal siMET vs. basal control samples.
